# Society Meetings

**Published:** 1901-03

**Authors:** 


### SOCIETY MEETINGS.

THE Third Pan-American Medical Congress was held at Havana, February 4-8, 1901, under the presidency of Dr. Juan Santos Fernandez, of Havana. Governor-General Wood welcomed the congress in appropriate terms at the Tacon Theatre, February 4th, in the presence of a brilliant audience. About 400 members were in attendance at the sessions, 100 of whom were from the United States. The Congress seems to have been a scientific success. The next meeting will be held at Buenos Aires in 1903.

---

THE Niagara Falls Academy of Medicine held its regular meeting at the office of Dr. C. G. Leo-Wolf, Monday evening, February 4, 1901, at which time Dr. J. H. Miller read a paper on Fever, its cause and treatment.

---

THE American Congress of Tuberculosis will hold its second annual meeting at the Grand Central Palace, in the City of New York, on the 15th and 16th days of May, 1901, in joint session with the Medico-Legal Society of New York.

THE Buffalo Academy of Medicine held meetings during the month of February, 1901, as follows:

*Section on Surgery.*—Tuesday evening, February 5th. Program: Conservative surgery of the extremity, Chauncey Pelton Smith. Surgical complications of typhoid fever, Vertner Kenerson. Operative treatment of tubercular lymphomata of the neck, Prescott LeBreton.

*Section on Medicine.*—Tuesday evening, February 12th. Program: The value of clinical pathology, A. E. Woehnert; Pemphigus, with the record of a case, J. W. Grosvenor; discussion by Grover Wende. Report of a case of psychical equivalent of epilepsy, Charles Cary and Julius Ullman.

*Section on Ophthalmology, Otology, Rhinology and Laryngology.*—Monday evening, February 18th. Program: Presentation of a case—macular choroiditis, with cholesterin crystals, J. C. Clemesha; The eye in nervous diseases, W. C. Krauss; Certain diseases of the eye benefited by treatment of the nose and naso-pharynx, B. H. Grove.

*Section on Pathology.*—Tuesday evening, February 19. Program: Leukemia, Chas. S. Jewett. House distribution of cancer in Buffalo during the past twenty years, Irving P. Lyon. Formaldehyde gas—its most simple application and its limitation in household disinfection, William G. Bissell.

*Section on Obstetrics.*—Tuesday evening, February 26th. Program: The toxemia of pregnancy, M. A. Crockett; Symphysiotomy, P. W. VanPeyma.

---

THE Society of Physicians of Canandaigua has issued its program for 1901, which is as follows:

February 7th, A. L. Beahan, Diagnosis of right side abdominal disease; March 7th, O. J. Hallenbeck, Spinal Curvature; April 4th, W. B. Clapper, Tetanus with report of three cases; May 2d, J. H. Jewett; June 6th, J. H. Pratt, Chronic gonorrheal inflammation of the uterine appendages; September 5th, D. A. Eiseline, Rheumatic myocarditis; October 3d, D. R. Burrell, Mental derangement in lower animals; November 7th, F. E. McClellan, Prevention and management of infection of the breast during lactation; December 5th, H. C. Buell, A low specific gravity.

At the annual meeting January 2, 1902, the subject of the address by the retiring president, Dr. M. R. Carson, will be The care of feeble children.

The officers are: President, M. R. Carson; vice-president, A. L. Beahan; secretary and treasurer, H. C. Buell.

The Western Ophthalmologic and Oto-laryngologic Association will meet in its next annual session at Cincinnati, Ohio, April 11 and 12, 1901. Dr. C. R. Holmes, of Cincinnati, is chairman of the local committee of arrangements. Dr. M. A. Goldstein, of St. Louis, is the president, and Dr. W. L. Ballenger, of Chicago, is the secretary.

